# The Association of Physicians and Surgeons of Bell County

**Published:** 1898-06

**Authors:** 


### The Association of Physicians and Surgeons of Bell County.

This society was recently organized at Belton with the election of Dr. Taylor Hudson as president, and Dr. Wilson T. Davidson,* secretary and treasurer. It meets the third Tuesday in each month.

*Now Captain and Assistant Surgeon Third Texas Vol. Infantry, U. S. A.—ED.

